# Mouse Models of HIV-Associated Atherosclerosis

**DOI:** 10.3390/ijms26073417

**Published:** 2025-04-05

**Authors:** Victoria R. Stephens, Sharareh Ameli, Amy S. Major, Celestine N. Wanjalla

**Affiliations:** 1 Department of Medicine, Vanderbilt University Medical Center, Nashville, TN 37232, USA; s.ameli@vanderbilt.edu (S.A.); amy.major@vumc.org (A.S.M.); 2 Division of Infectious Diseases, Vanderbilt University Medical Center, Nashville, TN 37232, USA; 3 Department of Pathology, Microbiology and Immunology, Vanderbilt University Medical Center, Nashville, TN 37232, USA; 4 Division of Rheumatology and Immunology, Vanderbilt University Medical Center, Nashville, TN 37232, USA; 5 Tennessee Valley Health System, Department of Veterans Affairs, Nashville, TN 37212, USA

**Keywords:** HIV, mouse models, cardiovascular disease, atherosclerosis, inflammation

## Abstract

Cardiovascular disease (CVD) remains the leading cause of death worldwide. Several factors are implicated in the pathogenesis of CVD, and efforts have been made to reduce traditional risks, yet CVD remains a complex burden. Notably, people living with HIV (PLWH) are twice as likely to develop CVD compared to persons without HIV (PWoH). Intensive statin therapy, the first-line treatment to prevent cardiovascular events, is effective at reducing morbidity and mortality. However, statin therapy has not reduced the overall prevalence of CVD. Despite antiretroviral therapy (ART), and new guidelines for statin use, PLWH have persistent elevation of inflammatory markers, which is suggested to be a bigger driver of future cardiovascular events than low-density lipoprotein. Herein, we have summarized the development of atherosclerosis and highlighted mouse models of atherosclerosis in the presence and absence of HIV. Since most mouse strains have several mechanisms that are atheroprotective, researchers have developed mouse models to study CVD using dietary and genetic manipulations. In evaluating the current methodologies for studying HIV-associated atherosclerosis, we have detailed the benefits of integrating multi-omics analyses, genetic manipulations, and immune cell profiling within mouse models. These advanced approaches significantly enhance our capacity to address critical gaps in understanding the immune mechanisms driving CVD, including in the context of HIV.

## 1. Introduction

**Figure 1:**
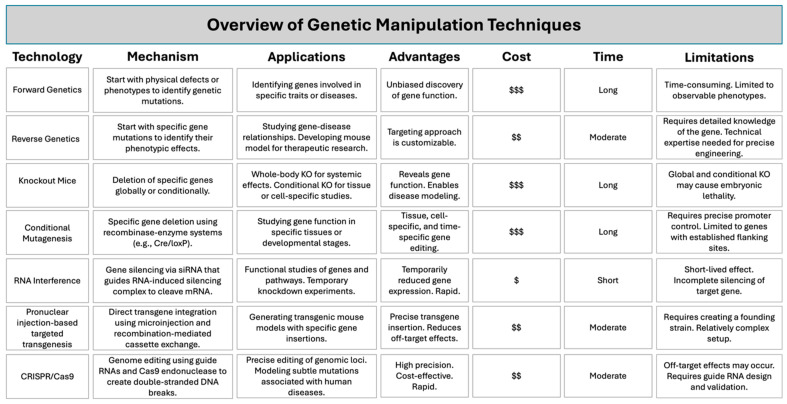
Overview of technologies used to modify mice for research. This table summarizes various genetic manipulation techniques, including their mechanisms, applications, advantages, cost, time, and limitations. Techniques covered include forward and reverse genetics [4], gene knockouts [5], conditional mutagenesis [6], RNA interference (RNAi) [7], pronuclear injection-based targeted transgenesis (PITT) [8], and CRISPR-Cas9 [9]. **$** = low cost, **$$** = moderate cost, **$$$** = high cost based on technical complexity and resource needs.

Mice, *Mus musculus*, are one of the most preferred model organisms in biomedical research for several reasons [1,2]. High genetic similarity to humans, rapid breeding, short gestation, and relatively large litter sizes make mouse models exceptionally useful for quickly generating and studying mechanisms of human disease. Although numerous scientific agencies prefer and encourage alternative experimental methods, mouse models have proven to be an invaluable tool for insights into human disease. In fact, the use of mouse models has increased due to advancements in genome-editing techniques, fostering the development of unique, genetically engineered mouse strains (Figure 1) [3]. Herein, we highlight advancements in mouse technologies and how they have been leveraged to benefit atherosclerotic cardiovascular disease (ASCVD) research. Mice mature significantly quicker than humans, allowing researchers to observe disease progression in real-time and at an accelerated rate. Notably, mice are relatively inexpensive, easy to handle, easy to house, have low maintenance costs, and are more suitable for high-throughput studies and non-invasive imaging than alternative animal models. In this review, we discuss how these technologies may be beneficial in developing mouse models for studying HIV-associated ASCVD and detail potential mechanisms for HIV-mediated atherogenesis in people living with HIV (PLWH).

## 2. Advancements in Mouse Model Technology

Mice and humans are roughly 90% genetically homologous with approximately 40% genomic alignment [10]. This conservation has supported genomic manipulations in mouse models to study human disease. Traditionally, these studies have used a ‘forward genetics’ approach, which involves identifying a physical defect or phenotype before investigating the underlying genetic mutation [3]. In recent years, researchers have opted to employ ‘reverse genetics’, allowing researchers to purposefully introduce genetic mutations and study the resulting phenotype(s). The most common example is the knockout (KO) mouse. KO mice were originally generated by deleting genes in embryonic stem cells and injecting the modified cells into blastocysts [11,12]. Blastocysts were subsequently implanted into pseudo-pregnant mice, and the resulting embryos would develop into offspring harboring the pre-engineered genome, representing whole-body KO mice. While whole-body KO models are ideal for some studies, the global deletion of some genes can be embryonically lethal [13,14,15,16]. Whole-body KO models also make it difficult to draw conclusions about the specific role of genes in individual cell types.

To overcome this limitation, researchers developed conditional mutagenesis, which transforms functional alleles into mutant alleles using recombinase-enzyme-target sequence systems, including Cre/loxP. This mutation can occur at a specific time (inducible conditional mutagenesis) or in a specific cell/tissue type (tissue-specific conditional mutagenesis) [3]. These systems work by flanking the target region of a gene with palindromic recombinase target sequences that recombine using a recombinase enzyme. When the target sequence recombines, the coding exon of the target gene undergoes an inversion or deletion, resulting in a genetic mutation. In the absence of the recombinase, gene expression remains unaffected, hence the ‘conditionality’ of this approach.

RNA interference (RNAi) is another tool used to modify gene expression in mice [17]. This technique requires double-stranded (ds) RNA homologous to the messenger RNA (mRNA) of the target gene. The dsRNA is recognized and fragmented into small interfering RNAs (siRNAs) by an enzyme called Dicer. These siRNAs are then integrated into the RNA-induced silencing complex (RISC). The siRNAs guide RISC to the mRNA of the target gene, where it binds and cleaves the mRNA, resulting in gene silencing. This technology was first introduced in the nematode *Caenorhabditis elegans* and later developed as a ‘knockdown’ method in mice [18,19].

Pronuclear injection-based targeted transgenesis (PITT) is another technique developed to modify genes [20]. This technology enables the integration of a single, complete copy of a transgene at specific genomic loci in a zygote by microinjecting directly into the pronuclei. The PITT method consists of two steps: (1) inserting a landing pad (i.e., target expression cassettes) at the target locus in the genome of embryonic stem cells to create the founding mouse strain, then (2) injecting the PITT components (a Cre source and a donor plasmid containing the DNA of interest (DOI)) into fertilized eggs harvested from the founding mouse. Since the landing pad and the DOI contain homologous sequences, the DNA is incorporated into the target locus using recombination-mediated cassette exchange (RMCE). This technology alleviates off-target transgene insertions and reduces the timeframe associated with previous methods.

Programmable endonucleases for genome editing are among the latest and most popular technologies used in mouse modeling. Programmable endonucleases act by causing non-homologous end joining (NHEJ; error-prone DNA repair) or homology-direct repair (precise DNA repair) to introduce genetic mutations. The clustered regularly interspaced short palindromic repeats/CRISPR-associated 9 (CRISPR/Cas9) system is the most preferred of the programmable endonucleases (i.e., homing endonucleases (HEs)), transcription activator-like effector nucleases (TALENs), and zinc-finger nucleases (ZFNs)). Endonuclease systems allow DNA to be cleaved at specific sites within the genome. CRISPR/Cas9 utilizes guide RNAs complementary to the target genes, which directs the endonuclease, Cas9, to the desired site where it makes a double-stranded break in the DNA [9]. CRISPR/Cas9 has ranked superior to the other systems due to its efficiency, cost-effectiveness, simplicity, and remarkable and steady improvements [21]. Since many human diseases result from subtle changes in the genome, using a precise and rapid genomic editing tool such as CRISPR/Cas9 has nearly made the other technologies obsolete in models of human disease.

## 3. Advantages of Using Mouse Models in Atherosclerotic Cardiovascular Disease Research

**Figure 2:**
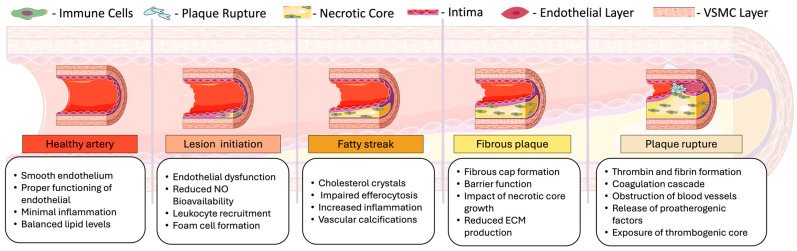
Stages of Atheroma Formation. Atherosclerosis develops in sequential stages, starting from endothelial dysfunction to plaque rupture. Healthy Artery: The endothelium functions to maintain minimal inflammation, balance lipid levels, and a healthy vascular environment. Lesion Initiation: Endothelial cells (ECs), which line the blood vessel walls, lose their integrity, leading to increased vasoconstriction, lipid infiltration, leukocyte adhesion, and oxidative stress. The reduced bioavailability of nitric oxide (NO), a key vasoprotective molecule, is a hallmark of endothelial dysfunction. This stage sets the foundation for plaque development. Fatty Streak: Pro-atherogenic mediators, such as cytokines and adhesion molecules, recruit monocytes to the intima. These monocytes differentiate into macrophages, internalizing low-density lipoproteins (LDL) and forming foam cells, initiating fatty streak formation. Fibrous Plaque: A fibrous cap forms over the necrotic core, composed of collagen and other extracellular matrix (ECM) components, stabilizing the plaque. However, the fibrous cap can weaken due to vascular smooth muscle cell (VSMC) apoptosis and the release of matrix metalloproteinases. Plaque Rupture: When the fibrous cap ruptures due to hemodynamic forces, platelets aggregate, and a coagulation cascade leads to thrombus formation. This exacerbates the plaque, potentially obstructing blood flow and contributing to cardiovascular disease. Servier Medical Art was used to create the figure, which is licensed under CC BY 4.0 (https://creativecommons.org/licenses/by/4.0/), assessed on January 30 2023.

CVD (i.e., stroke, ischemic heart disease, and peripheral vascular disease) is the leading cause of death worldwide, and atherosclerosis is the primary risk factor for CVD. Atherosclerosis is characterized as a chronic inflammatory disease resulting in the accumulation of lipids, fibrous elements, immune cells, and calcification—collectively known as plaque—within arterial walls (Figure 2) [22]. While wild-type mice do not spontaneously develop atherosclerotic lesions, knockout and transgenic mice have been instrumental in studying the development, progression, and cellular and molecular mechanisms involved in atherosclerosis. Mouse models have also been advantageous in examining the efficacy of anti-atherogenic therapies. Leading mouse models in atherosclerotic research primarily rely on genetic modifications that alter lipoprotein metabolism. These animal models can be further manipulated through dietary changes, promoting the development of additional risk factors for cardiovascular disease, including diabetes and hypertension. 

## 4. Mouse Models for Atherosclerosis

Atherosclerosis is an immunometabolic disease involving multiple organs, cell types, and processes, and they do not exhibit complete homology between humans and mice. As a result, not all mouse models can fully recapitulate all aspects of ASCVD. Researchers have developed several mouse models using dietary and genetic manipulations that exhibit diverse characteristics of ASCVD [23]. These models allow for the investigation of various facets of atherogenic processes, including cholesterol metabolism, lipoprotein metabolism, immunometabolic regulations, and inflammatory processes.

Elevated plasma LDL is a primary risk factor for atherosclerotic development and disease progression. Unlike humans, mice primarily transport cholesterol in high-density lipoprotein (HDL) particles, facilitating cholesterol removal from the bloodstream. This lipid profile contributes to their relative resistance to atherosclerosis [24]. Mice also synthesize and produce more hydrophilic bile acids than humans [25,26]. Therefore, mice have reduced intestinal cholesterol uptake with increased fecal cholesterol excretions, further highlighting atheroprotective mechanisms in mice. Researchers have targeted various genes to make mouse models more prone to developing atherosclerosis, making transgenic mice a common model organism for studying ASCVD.

Laboratory mice are typically fed a standard chow diet containing 0.03% cholesterol and 6% fat, which does not promote atherogenesis. However, humanized diets, such as the Western-type diet and other atherogenic diets, can induce atherogenesis in mouse models genetically prone to developing atherosclerosis [27]. Dietary changes rarely promote the development of atherosclerosis in wild-type mice; however, some mouse strains are more prone to develop CVD than others. For example, C57BL/6 mice have lower HDL levels compared to BALB/c mice, which increases their susceptibility to atherogenesis, especially on a humanized, high-fat diet [28]. C57BL/6 mice are also more susceptible to lesion formation on an atherogenic diet (1.25% cholesterol, 0.5% cholic acid, and 15% fat) compared to 9 other commonly used mouse strains [29]. C57BL/6 mice are also more prone to obesity and diabetes, which are well-known risk factors for atherosclerosis [30]. In addition to their lipoprotein metabolism, mice on a C57Bl/6 background are an ideal model for studying atherogenic processes due to their Th1-skewed immune profile compared to other mouse strains. T cells in C57BL/6 mice primarily produce Th1 cytokines such as interferon (IFN)-γ, which has been pivotal in atherosclerotic lesion development and plaque rupture in atherosclerosis [31].

Although C57BL/6 mice are more pro-atherogenic than other mouse strains, researchers have further manipulated them to increase their susceptibility to atherosclerotic disease. One approach involves deleting the gene responsible for expressing apolipoprotein E (ApoE). The deletion of *Apoe* fosters the accumulation of cholesterol in plasma, resulting in hypercholesterolemia. With the addition of a high-fat diet, *Apoe^−/−^* mice possess cholesterol levels greater than 1000 mg/dL compared to the 400–600 mg/dL observed in mice on a standard chow diet [32]. *Apoe^−/−^* mice also express elevated levels of endothelial cell adhesion molecules, triggering leukocyte recruitment and transmigration of monocytes into the intima [33]. The absence of ApoE increases the proliferation and motility of vascular smooth muscle cells (VSMCs) and promotes platelet aggregation. Furthermore, *Apoe^−/−^* mice display a high degree of impaired efferocytosis, increasing the accumulation of apoptotic cells in the vessel wall [23]. Together, these characterizations highlight the pro-atherogenic features of this mouse model.

In contrast to humans, *Apoe*^−/−^ mice develop advanced-stage atherosclerotic plaques that rarely rupture, eliminating the potential for investigating the thrombogenic phenotypes observed in human disease. Another limitation of this model stems from the difference in their lipoprotein profile compared to humans [23]. *Apoe^−/−^* mice shuttle their plasma cholesterol using chylomicron particles and very low-density lipoprotein (VLDL) particles, whereas humans primarily use LDL particles. To combat this limitation, Westerterp et al. crossbred ApoE*3-Leiden (E3L) mice with human *cholesteryl ester transfer protein* (*CETP*) transgenic mice to investigate the role of CETP in atherogenesis. E3L transgenic mice exhibit hyperlipoproteinemia [34]. However, when crossbred with *CETP* transgenic mice, the expression of VLDL/LDL-cholesterol is increased [35]. These CETP.E3L mice exhibit advanced-stage atherosclerotic lesion development characterized by the enlarged lesion area, calcifications, and cholesterol clefts. Due to its similarity to humans, this model is particularly useful for studying atherogenic processes, especially regarding age-related changes in cholesterol transport and lipid profiles.

Another commonly used mouse model for studying atherosclerosis is the low-density lipoprotein receptor (*Ldlr*) knockout mouse. These mice are suggested to be a closer replica of human disease than *Apoe^−/−^* mice since most cholesterol is transported by LDL particles in the plasma [23]. LDLR is a membrane-bound receptor that mediates the endocytosis of LDL in plasma. In its absence, cholesterol levels rise as high as 300 mg/dL in mice on a standard chow diet and approximately 1000 mg/dL in mice on an atherogenic diet [36]. Interestingly, studies have revealed over 600 genetic mutations in human *Ldlr*, many of which have been linked to familial hypercholesterolemia and the subsequent development of atherosclerosis [23,37].

While the mouse models described above are sufficient for answering specific questions regarding atherogenic processes, as highlighted in Table 1, they are limited because the presentation and location of atherosclerotic plaques differ between mice and humans. In humans, plaques typically develop in the carotid and coronary arteries. Mice usually develop plaques in the brachiocephalic trunk, aortic arch, aortic sinus, and proximal aorta. Furthermore, in humans, atherosclerotic plaques develop into fibrous atheroma, while mice do not develop advanced-stage atheromatous disease. Herck et al. created a mouse model that develops atherosclerotic plaques markedly similar to late-stage human disease [38]. In addition to the characteristics described in the *Apoe*-deficient mice, these *Apoe^−/−^ Fibrillin 1* (*Fbn1*)^C1039G+/−^ mice contain a mutation in the *Fbn1* gene that contributes to elastin fragmentation, a pivotal contributor to arterial stiffening, atherosclerotic plaque development, plaque instability, and plaque rupture. Similarly, plaque destabilization and rupture have also been observed in *Apoe^−/−^* mice on a high-fat diet for four weeks, followed by an infusion of angiotensin II for four weeks [39]. Angiotensin II increases leukocyte recruitment, activation, and oxidative stress, which are critical for the development and progression of atherosclerosis, making this model ideal for studying atherogenesis. While mice are advantageous for the study of atherosclerosis, there are several limitations as highlighted in Table 2.

## 5. Mouse Models of HIV-Associated Atherosclerosis

ASCVD has increasingly become a common cause of morbidity and mortality in PLWH. Unfortunately, determining whether this phenomenon is due to HIV-associated inflammation, the use of combination antiretroviral therapy (cART), or other risk factors remains an enigma. Researchers have created transgenic and chimeric mouse models of HIV-associated atherosclerosis, enabling the investigation of co-occurring diseases.

The Tg26 mouse model is a transgenic strain that expresses HIV transcripts without active viral replication [40]. However, these mice still develop HIV-related comorbidities, such as atherosclerosis, indicating that the presence of HIV transcripts and proteins alone can contribute to atherogenesis and other co-occurring conditions. Using Tg26 mice, Kress and colleagues reported that HIV-infected CD4^+^ T cells increase IL-1α production, which increases NOX1, leading to impaired endothelial function [41]. To model HIV-associated atherogenesis, Alam et al. crossed Tg26 mice with *Apoe^−/−^* mice (Tg26^+/−^/*Apoe^−/−^* mice) [42]. After being on a high-fat, high-cholesterol (atherogenic) diet for 8 weeks, data revealed that these mice had accelerated atherosclerosis compared to *Apoe^−^*^/*−*^ mice, as indicated by the significant increase in atherosclerotic plaques. Tg26^+/−^/*Apoe^−/−^* mice were also found to increase monocytic caspase-1 activation. HIV-associated inflammation has been well-documented in its ability to activate the NLRP3/caspase-1 inflammasome [43]. Interestingly, Alam et al. found that caspase-1 deficiency in Tg26^+/−^/*Apoe^−/−^* mice attenuated the HIV-associated atherosclerotic phenotypes previously observed (i.e., increased plaque burden and macrophage infiltration) [42]. The serum kynurenine to tryptophan ratio (KTR) is a biomarker of inflammation that has been linked to an increased risk for the development of cardiovascular disease in cART-treated patients with HIV. Kearns and colleagues have found that Tg26^+/−^/*Apoe^−/−^* mice increased KTR levels compared to Tg26^+/−^ and *Apoe*^−/−^ single transgenic mice [44]. Collectively, these data suggest that Tg26^+/−^/*Apoe^−/−^* transgenic mice serve as an insightful model for investigating HIV-induced atherosclerosis.

Since single-protein transgenic mice do not develop atherosclerotic plaques, researchers have used these mouse models to examine specific and isolated components of atherogenesis, such as cholesterol metabolism. HIV has been shown to impair cholesterol metabolism through downstream functions of Nef, an HIV accessory protein. ABCA1 is a cellular cholesterol transporter; however, when bound by Nef, the expression of ABCA1 is downregulated, leading to lipid accumulation in macrophages and their conversion to foam cells, a hallmark of atherosclerosis [45]. To examine this mechanism in vivo, Cui et al. fed *Apoe^−^*^/*−*^ and C57Bl/6 mice a high-fat, high-cholesterol diet before injecting them with recombinant Nef [46]. Data from this study revealed a significant increase in atherosclerotic plaques in *Apoe*-deficient mice compared to C57BL/6 mice. They also found that these animals had increased plasma cholesterol and triglyceride levels, which was associated with reduced ABCA1 expression in the liver. Interestingly, C57BL/6 mice injected with recombinant Nef had a significantly increased accumulation of lipid-laden macrophages in the adventitia of the aortic vessels compared to C57BL/6 mice injected with saline (vehicle). Additionally, Pushkarsky and colleagues observed an increased accumulation of neutral lipids in the aorta of Nef-transgenic mice compared to control mice [47]. Taken together, these data suggest that HIV proteins, specifically Nef, are sufficient for inducing HIV-associated atherogenesis.

## 6. Atherosclerotic Cardiovascular Disease in People Living with HIV

### 6.1. Direct Effects of HIV on Vascular Cells

**Figure 3:**
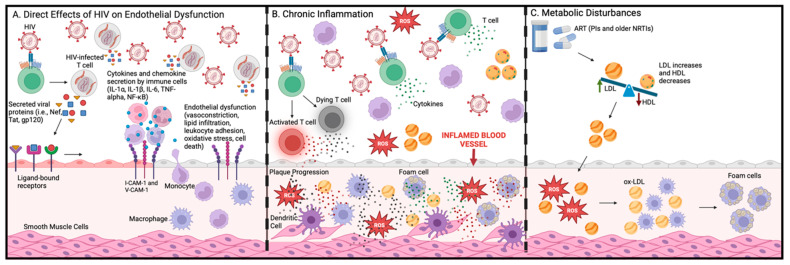
Direct effect of HIV on endothelial dysfunction. (**A**) HIV binds to the CD4 receptor on the surface of a T cell, fuses with the T cell, and replicates inside the T cell, HIV-secreted viral proteins, such as Nef and Tat, leading to the upregulation of adhesion molecules like ICAM-1 and VCAM-1. Additionally, immune cells secrete pro-inflammatory cytokines and chemokines, including IL-1α, IL-1β, IL-6, TNF-α, and NF-κB, further contributing to endothelial dysfunction. This dysfunction results in pathological outcomes such as vasoconstriction, lipid infiltration, leukocyte adhesion, oxidative stress, and cell death. (**B**) Chronic inflammation begins when a virus recognized by the T-cell receptor (TCR) causes T-cell death. These dead cells produce more pro-inflammatory cytokines, increasing reactive oxygen species (ROS) and oxidative stress. In endothelial cells, the early stages of atherosclerosis are marked by LDL entering the blood vessel wall, which becomes oxidized and induces monocyte recruitment. Recruited monocytes differentiate into macrophages, taking up oxLDL via scavenger receptors. This scavenger-receptor-mediated uptake of lipoproteins by macrophages leads to the formation of foam cells. (**C**) ART is a class of medications that suppress viral replication in HIV infections. As a side effect, some inhibitors can cause an imbalance between LDL and HDL levels, increase ROS production, and disrupt the body’s ability to neutralize this production damaging molecules. This results in more oxidative stress, increased oxLDL, the formation of more foam cells, and promotion of plaque formation. Created with BioRender.com, accessed 19 February 2025.

Although HIV cannot undergo active replication in endothelial cells (ECs), HIV-encoded proteins released into the microenvironment or transferred onto ECs during an HIV infection can directly activate ECs and compromise the integrity of the endothelium (Figure 3) [41,48].

### 6.2. HIV Glycoprotein (gp 120)

Gp120 is expressed on the outer layer of HIV particles and the surface of infected cells, and it facilitates viral entry and binding specificity on target cells. Studies have shown that gp120 can directly upregulate pro-atherogenic cytokines (IL-6 and IL-8) and cell adhesion molecules (CAMs) to facilitate leukocyte recruitment and adherence to the vascular endothelium, respectively [49,50]. Gp120 also increases endothelial cell permeability and induces the expression of matrix metalloproteinase-9, which contributes to endothelial dysfunction by degrading extracellular matrix-associated proteins [51,52,53,54].

### 6.3. HIV Trans-Activator of Transcription (Tat)

Tat is secreted into the extracellular micromilieu by HIV-infected monocytes, macrophages, and T-cells, enhancing viral transcription. Tat also elicits endothelial dysfunction by activating integrins (Vbeta1, Vbeta3, and alphaVbeta5) to initiate pro-atherogenic signaling cascades in the vascular endothelium [55,56]. Studies have shown that Tat can induce EC apoptosis through cytokine secretion and the activation of Fas-dependent cell signaling pathways [57]. Like gp120, Tat also upregulates the production of pro-inflammatory cytokines and adhesion molecules [58,59]. Specifically, Tat stimulates the secretion of IL-β which can potentially promote apoptosis in macrophages and foam cells, directly contributing to the growth of the lipid-rich necrotic core. MCP-1 secretion is also upregulated in the presence of Tat, facilitating the transendothelial migration of monocytes from the plasma to the intima [60]. Subsequently, these monocytes are differentiated into macrophages, potentially becoming foam cells, a significant component of the necrotic core.

### 6.4. HIV Negative Regulatory Factor (Nef)

Nef is an accessory protein that aids in increasing the infectivity of HIV. Nef induces endothelial dysfunction by inducing apoptosis via ROS-dependent mechanisms and upregulating MCP-1 via NF-kB [61]. Furthermore, Duffy et al. revealed that Nef decreases nitric oxide production through the inhibition of adequate endothelial nitric oxide synthase (eNOS) expression, which, in turn, reduces endothelium-dependent vasodilation, a hallmark sign of atherosclerosis [62]. Like gp120 and Tat, Nef has also been shown to induce apoptosis in ECs through several mechanisms, including mitochondrial pathways, Fas/FasL-dependent pathways, and the activation of caspases [61,63,64]. Nef also increases the production of MCP-1, ultimately increasing the potential for foam cell formation and the development/progression of atherosclerosis [65,66].

### 6.5. Inflammation and Immune Activation

**Figure 4:**
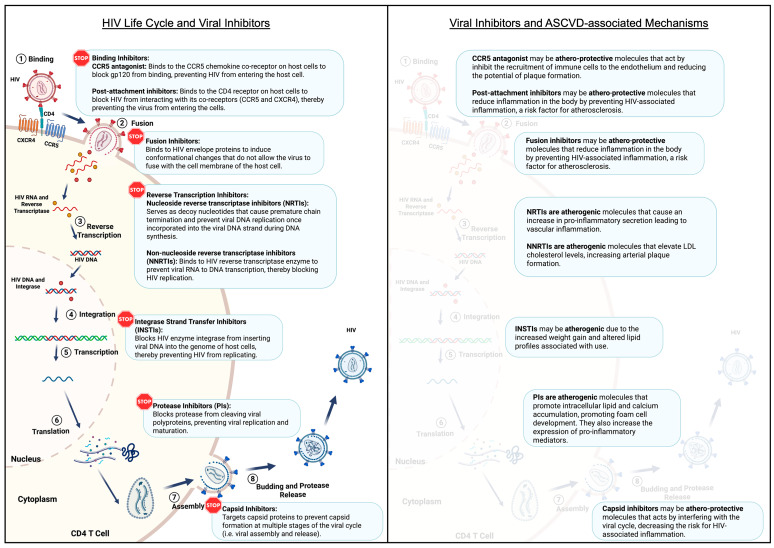
HIV life cycle, viral inhibitors, and ASCVD-associated mechanisms. (**Left Panel**) This figure illustrates the stages of the HIV life cycle, including binding and post-attachment, viral entry, reverse transcription, integration, transcription, translation, and budding. It also highlights the points at which various classes of antiretroviral drugs act to disrupt the viral replication process, including binding inhibitors (CCR4 antagonist), entry inhibitors, nucleoside reverse transcriptase inhibitors (NRTIs), non-nucleoside reverse transcriptase inhibitors (NNRTIs), integrase strand transfer inhibitors (INSTIs), protease inhibitors (PIs), and capsid inhibitors. (**Right Panel**) Among these viral inhibitors, CCR4 antagonists, post-attachment, fusion, and capsid inhibitors are atheroprotective. In contrast, nucleoside reverse transcriptase inhibitors (NRTIs), non-nucleoside reverse transcriptase inhibitors (NNRTIs), integrase strand transfer inhibitors (INSTIs), and protease inhibitors (PIs) may be atherogenic. Created with BioRender.com, accessed on 28 January 2025.

While ART can effectively suppress viral replication, aviremic PLWH continue to experience residual and chronic inflammation associated with the virus (Figure 4). Persistent inflammation is associated with an increased risk for multiple comorbidities, including atherosclerosis. Several immune cells affected by HIV also contribute significantly to the development and progression of ASCVD. For example, macrophages are a cellular reservoir for latent HIV. Andrade et al. suggest that macrophages are infected early during the acute phase of the HIV infection due to the identification of rebound HIV particles with macrophage-specific markers on their surface (macrophage-tropic (M-tropic) virus) [67]. Since the M-tropic viruses were identified in viremia from PLWH during treatment interruption, these data suggest that macrophage reservoirs can reignite viremia and contribute to viral persistence. Upon infection, macrophages shift to an M1 phenotype, leading to the expression and secretion of pro-inflammatory cytokines such as IFN-γ. HIV infection also upregulates anti-apoptotic genes in macrophages through various pathways (including NF-kβ/TREM1 and PI3K survival pathway) to promote their survival [68,69,70,71].

Many macrophage-associated inflammatory pathways that contribute to viral persistence are also key drivers of atherogenesis and atherogenic processes. As previously mentioned, HIV promotes macrophage survival by increasing the expression of BCL2, MFN1, MFN2, and BCLXL and decreasing the expression of BAX and BAD [71]. The increase in the number of surviving macrophages potentially fosters the increased recruitment of macrophages into the intima. This, in turn, leads to more cellular uptake of oxidized LDLs (oxLDLs) and the transformation of macrophages into foam cells, which are essential for the initiation, formation, and growth of atherosclerotic lesions. On the other hand, some HIV-infected macrophages undergo apoptosis [72]. This suggests that increased accumulation of apoptotic macrophages and impaired efferocytosis could exacerbate atherosclerosis by enlarging the necrotic core. Furthermore, the increased production of IFN-γ during an HIV infection can promote pro-atherogenic processes by regulating cholesterol accumulation, foam cell formation, plaque formation, and plaque rupture. IFN-γ also stimulates macrophages to produce pro-inflammatory cytokines, contributing to chronic inflammation and increased expression of adhesion molecules, as seen in atherosclerosis [73].

**Figure 5:**
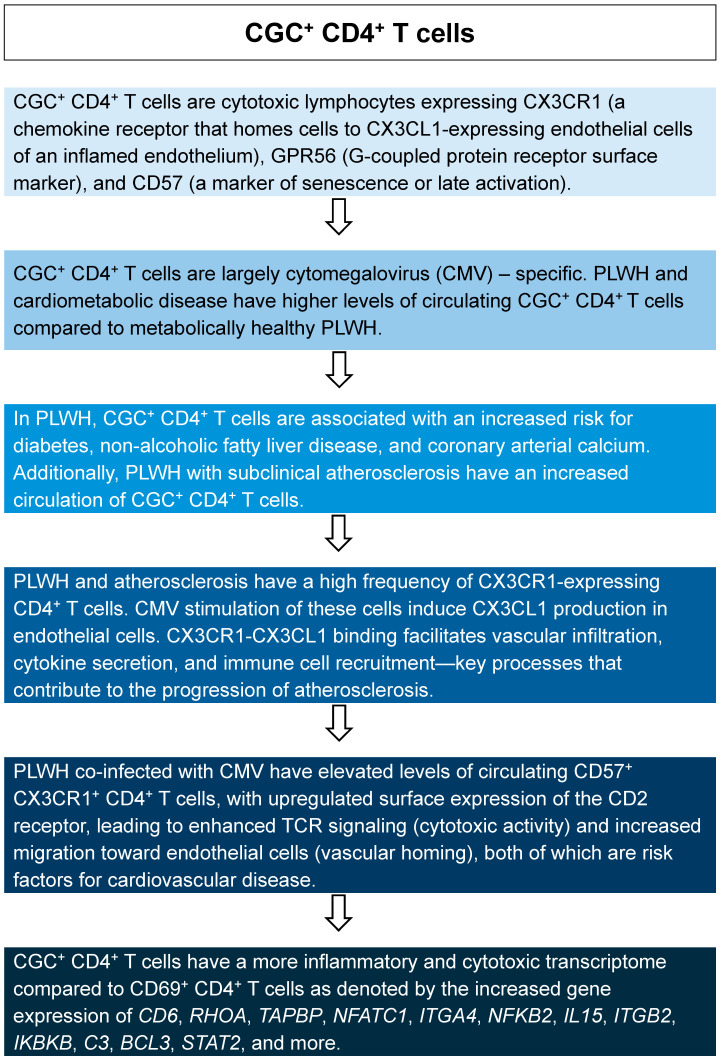
CGC^+^ CD4^+^ T cells. This figure summarizes the main findings on the role of CGC^+^ CD4^+^ T cells in cardiometabolic disease among PLWH [74,75,76,88,89].

In addition to macrophages, other immune cells and inflammatory components have also been linked to HIV-induced atherosclerosis (Table 3). Among the different subsets, our group has studied a specific CD4^+^ T cell subset in the context of HIV and cardiometabolic disease. These CD4^+^ T cells are identified by their co-expression of three surface markers: CX3CR1, GPR56, and CD57, referred to as CGC^+^ CD4^+^ T cells (Figure 5). Studies have shown that CGC^+^ CD4^+^ T cells are higher in PLWH than PWoH with diabetes and are associated with subclinical atherosclerosis [74,75,76,77].

**Table 3:** Summary of immune cells and inflammatory components linked to HIV-induced atherosclerosis.

PBMCs	Role in HIV	Pro-atherogenic Features and Mechanism of Atherogenesis	Ref.
MDMs	Monocytes and macrophages are reservoirs for HIV. HIV infection of these cells contributes to persistent inflammation.	Chronic inflammation contributes to endothelial dysfunction and adhesion, vascular inflammation, and plaque instability. HIV induces impaired lipid processing and metabolism in these cells. These mechanisms increase trans-endothelial migration, arterial accumulation, and cholesterol internalization (foam cell formation).	[78,79]
DCs	DCs play an important role in viral transmission to CD4^+^ T cells. HIV also increases the activation of DCs, promoting inflammation.	Activated DCs have increased antigen-presenting capabilities, allowing for increased presentation of ox-LDL to T-cells. This leads to heightened inflammation within the arterial wall. DCs also produce pro-inflammatory cytokines that recruit and activate other immune cells, promoting plaque development and rupture.	[80,81,82]
CD4^+^ T cells	HIV targets CD4^+^ T cells and uses its machinery to replicate. This causes the cells to die, leading to low CD4^+^ T cell counts without ART.	Low CD4^+^ T cell counts disrupt the balance between pro- and anti-inflammatory signaling. The increased inflammation promotes endothelial activation and dysfunction, promoting plaque formation and ASCVD progression.	[83,84]
B cells	HIV viral proteins and virions trigger the production of pro-inflammatory cytokines from B cells and increase apoptosis. HIV also alters their class switching capabilities.	B cells produce antibodies against ox-LDL and cytokines, likely promoting immune complexes that increase inflammation within the arterial wall. There are no previous studies of B cells in HIV-associated atherosclerosis.	[85]
NK cells	NK cells are crucial for the body’s defense against viruses. However, HIV can impair the antiviral properties of NK cells and inhibit their ability to interact with other immune cells, leading to increased production of inflammatory mediators.	NK cells are primarily in the necrotic core of atherosclerotic lesions. They produce IFN-γ, granzyme B, and perforin, which can contribute to enlarging the necrotic core and destabilizing the plaque, leading to plaque rupture.	[86,87]

Abbreviations: peripheral blood mononuclear cells (PBMCs); monocytes and monocyte-derived macrophages (MDMs); dendritic cells (DCs); natural killer (NK) cells.

### 6.6. Insights into Pathogenic Mechanisms

HIV-associated atherosclerosis is an active area of investigation. Several studies have highlighted how the interaction between HIV, immune cells, and ECs drives atherogenesis [48]. Oxidative stress, identified by elevated levels of reactive oxygen species (ROS), plays a major role in the development of atherosclerosis [90]. ROS is largely produced by nicotinamide adenine dinucleotide phosphate (NADPH) oxidase (NOX) enzymes, which are abundant in phagocytic leukocytes (i.e., macrophages) and highly expressed in smooth muscle cells and ECs. During hyperlipidemia, NOXs are upregulated and contribute to the increased formation of oxLDL. Cells internalize oxLDL, which undergo lysosomal degradation, and their downstream products mediate inflammatory signaling cascades that induce atherogenic processes (i.e., EC activation, chemokine expression, monocyte/macrophage recruitment, leukocyte recruitment and foam cell formation).

Using models of HIV and patient data, studies have shown that the presence of HIV correlates with elevated levels of ROS and oxLDL, likely due to enhanced production and impaired antioxidant functions [91]. Furthermore, Nef has been shown to increase ROS production in arteries and arterial ECs, contributing to endothelial dysfunction [62]. These data suggest that HIV-induced oxidative stress has the potential to drive atherogenic processes, leading to the development of atherosclerosis.

Increased ROS production following an HIV infection can also trigger endoplasmic reticulum (ER) stress. ER stress subsequently activates the unfolded protein response (UPR) during atherosclerotic lesion formation [92]. ER stress has also been shown to induce macrophage apoptosis [93]. When apoptosis is enhanced, the increase in apoptotic cells can overwhelm phagocytes, leading to impaired efferocytosis, which contributes to the development of the necrotic core in atherosclerotic plaques, as previously mentioned [94,95].

The inflammasome is an integral component of the innate immune system, and it functions by responding to inflammatory stimuli, such as HIV. Since HIV cannot be cleared by the immune system, its persistence leads to prolonged and excessive activation of the inflammasome, which has been linked to atherosclerosis [96]. The accumulation of cholesterol crystals and oxLDL also stimulates NLRP3 inflammasome activation [97,98]. Inflammasome activation triggers caspase-1, which cleaves pro IL-1β and pro IL-18 into their mature forms to mediate inflammatory responses. Interestingly, studies have shown that caspase-1 activation increases rapidly during HIV infection, and it promotes the release of mature and biologically active IL-1β and IL-18, two atherogenic cytokines [99].

The transmigration and adhesion of monocytes to the subendothelial space, where they differentiate into macrophages, internalize oxLDL, and become foam cells, are important for the initiation of atherogenesis. This process is primarily regulated by chemokines, chemokine receptors, and adhesion molecules. In fact, inhibiting the activity of several chemokines and chemokine receptors has been shown to reduce the size of atherosclerotic lesions, highlighting the critical role these molecules play in atherogenesis [100,101]. During HIV infection, chemokines and their receptors are produced and expressed at heightened levels, suggesting that HIV may contribute to atherogenesis by upregulating the production of these molecules.

Autophagy is a highly regulated process in which cells degrade dysfunctional or abnormal cellular components and proteins, recycling them for use in essential cellular processes. During an HIV infection, the role of autophagy is cell-dependent and typically supports the survival of the virus. For example, dendritic cells (DCs) act by accumulating HIV particles and then migrating to lymph nodes where they present and transfer HIV to CD4^+^ T cells. Studies have shown that immature, resting DCs are readily infected with HIV, whereas mature, activated DCs appear to be resistant to infection (depending on cellular origin) [102]. Mature CD4^+^ T cells can be activated by TNF-α and poly (I:C) which have been shown to activate and upregulate autophagy [103]. However, Blanchet et al. demonstrated that the HIV-associated Env protein rapidly downregulated autophagy in DCs through the mTOR pathways [104]. This mechanism protects HIV from autophagy-mediated degradation and promotes increased DC to CD4^+^ T cell viral transmission and survival. This mechanism also increases inflammation, which can promote atherosclerosis.

### 6.7. Optimizing Immune-Deficient Mouse Models for Atherosclerosis and HIV Research Using Human Immune Cells

Atherosclerosis research in the context of human immune cell function and HIV requires advanced humanized mouse models that support robust PBMC and hematopoietic stem cell (HSC) engraftment, enable atherosclerosis progression, and facilitate HIV persistence within macrophage-rich vascular lesions. Standard NSG mice provide an excellent foundation for human immune cell engraftment but lack optimal myeloid lineage support and atherosclerosis susceptibility [105,106]. Recent models, including NSG-SGM3, NSG-MHC-DKO, and NOD *Apoe^−/^^−^ LDLr^−/^^−^*, offer improvements but require additional modifications to fully capitulate human immune responses in vascular disease [105,106,107].

### 6.8. Key Features of NSG-Based Mouse Models for the Study of Atherosclerosis Progression in HIV

**Figure 6:**
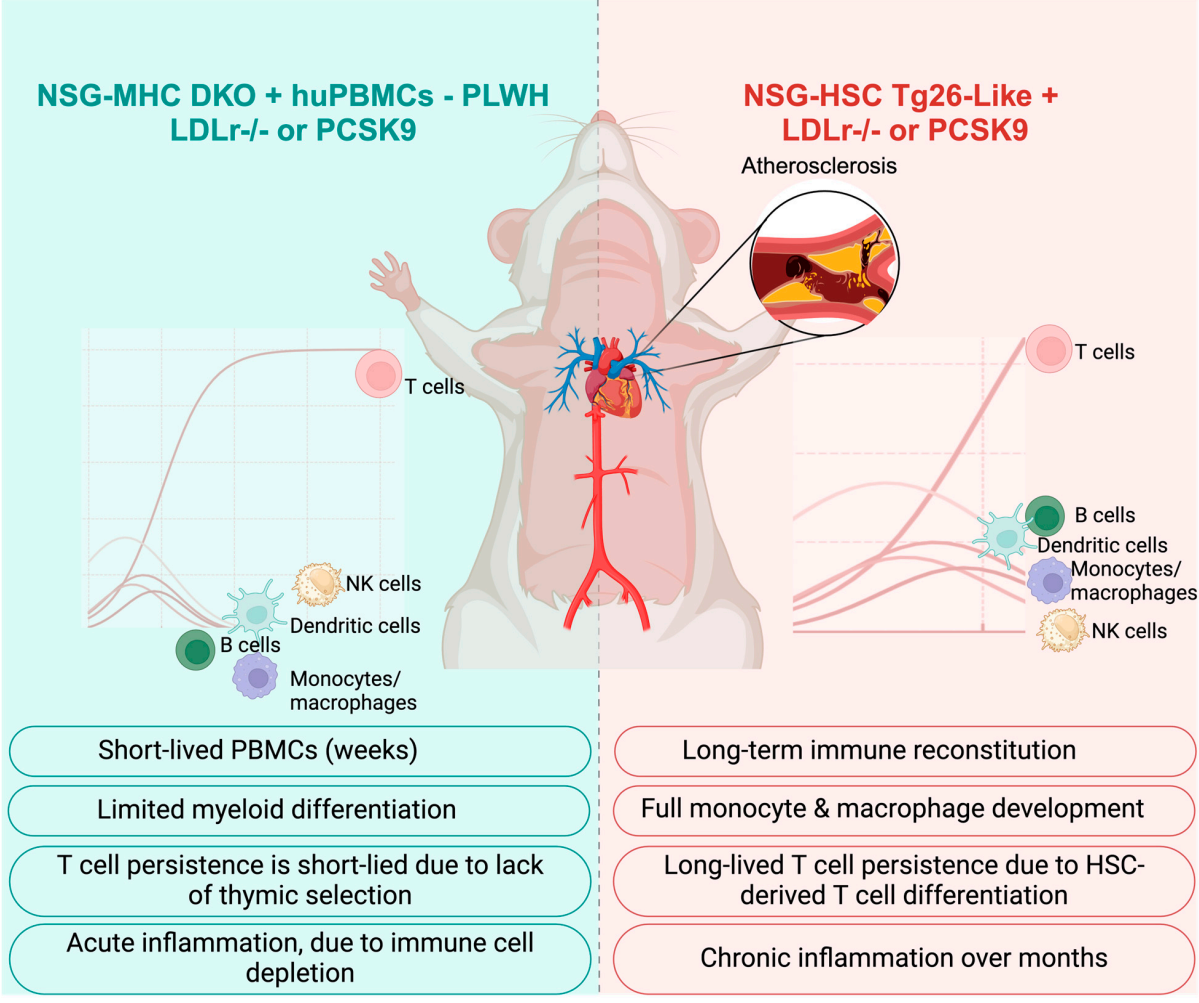
NSG-mouse models in CVD research related to HIV. On the (**left**), using NSG-MHC I/II double knock-out (DKO) mice reconstituted with PBMCs, the role of immune cells may be explored in the development stages of atherosclerosis. Due to the short-lived period, T cell functions will likely be better explored in this model. On the (**right)**, we propose that NSG mice reconstituted with human stem cells modified to express HIV proteins, like Tg26 mice, would be used. We propose knocking out the LDL receptor for both models because the cholesterol profile is like humans versus a gain of function *PCSK9* mouse, which can be performed using an adenoviral vector [105]. Created with BioRender.com, accessed on 16 February 2025.

The ideal mouse model would integrate human T cell and monocyte/macrophage function while allowing for severe atherosclerosis progression (Figure 6). For short-term studies, an optimized NSG-based mouse model would incorporate PBMC engraftment for T-cell studies and MHC Class I/II double knockout (MHC-DKO) to prevent xenogeneic GVHD, allowing human PBMC persistence. The mice would also need a genetic predisposition to atherosclerosis with either the *PCSK9* gain-of-function mutation to drive severe hypercholesterolemia or *LDLr^−/^^−^* to mimic HFD-induced atherosclerosis comparable to human cardiovascular disease in inflammatory settings. In addition, modifications of IL-7 and IL-15 transgenes to support human T cell and NK cell survival in vivo would support robust adaptive immune responses relevant to atherosclerosis and HIV persistence.

For long-term studies, NSG mice reconstituted with human stem cells will likely yield outcomes similar to those of humans. For HIV-related studies, we would leverage studies from Tg26 (HIV) mice and modify NSG mice to express transgenes with part of the HIV in the NSG mice or the HSC cells engrafted into the mice. Similar to the short-term studies, the genetic predisposition to atherosclerosis would be either the *PCSK9* gain-of-function mutation, *LDLr*^−/−^ and HFD, or a combination. Beyond T cells, enhanced myeloid and macrophage support through the expression of human CSF-1 would support macrophage development and survival following HSC engraftment. This would support HIV-infectible reservoirs in the vascular niche and promote antigen presentation. This idealized NSG-based model would integrate key genetic modifications to support patient-derived immune cells, ensure severe atherosclerosis progression, and provide a relevant platform for studying HIV-infected macrophages in vascular lesions. Further refinements in engraftment optimization, lipid metabolism modifications, and immune system fine-tuning will allow for greater translational relevance in CVD and HIV research.

## 7. Integration of Omics Approaches to Deepen Understanding

Omics refers to high throughput screening technologies, often providing unbiased molecular measurements within cells and tissues. These methods increase our understanding of biological systems beyond standard, non-computational experimental approaches. Examples of omics technologies include proteomics (global analyses of proteins), metabolomics (global analyses of metabolites), genomics (global analyses of genes), transcriptomics (global analyses of RNA), and more. Using omics data in biomedical research has been paramount in facilitating the exponential growth in medical advancements for more than a decade. These experimental tools provide data on differentially expressed molecules between typical and atypical biological pathways and processes, providing insight into potential disease biomarkers. Although each omics technology is powerful, no single technology can display the full scope of disease processes alone, which is why multi-omics is suggested.

Integrating multiple types of omics data and technologies (multi-omics) increases the potential of uncovering a comprehensive understanding of the pathogenic changes associated with disease. This can include identifying a link between biomolecules and disease or identifying up- and down-regulated signaling pathways relevant to disease phenotypes. A multi-omics approach allows researchers to uncover biological information efficiently and rapidly before verifying those findings using more traditional molecular approaches. As it relates to translational medicine and research, the application of multi-omics data generally has five main objectives: (1) detect disease-associated molecular patterns, (2) subtype identification, (3) diagnosis/prognosis, (4) drug response prediction, and (5) understand regulatory processes [108]. The multi-omics approach also provides an additional tool for validating the use and relevance of animal models, such as mouse models, to study human disease.

Atherosclerosis is an etiopathological factor of cardiovascular diseases, such as coronary artery disease (CAD). Kurt et al. conducted a comprehensive, integrative study using multiomics data resources, which allowed for a systems-level, tissue-specific analysis of CAD in mice and humans. They focused on genome-wide association studies (GWAS) to investigate genetic risk loci, transcriptomics to interrogate gene expression and patterns, and expression quantitative trait loci (eQTLs) to assess gene regulation in a tissue-specific manner [109]. This multi-layered, multi-omics approach revealed that mice and humans shared at least 75% of pathways that lead to CAD. Importantly, multi-omics approaches have been used to interrogate other known HIV-related CVD complications and risk factors, including metabolic syndrome, altered lipid profiles, atrial remodeling, and atrial fibrillation [110,111,112,113].

## 8. Conclusions

Mouse models have been used extensively to study mechanisms of atherosclerosis and to investigate and test therapeutic targets and drugs. Although more than 90% of protein-encoding genes are homologous between humans and mice, both species have distinct differences that impair direct translational discoveries. Differences in immune cell features between humans and mice are also notable. Furthermore, there are certain risk factors for atherosclerosis development, such as inflammation from HIV, that cannot be studied in most mouse models. Therefore, humanized mouse models are superior tools compared to the previously mentioned mouse models to study HIV-associated atherosclerosis. Using immunocompromised mice, such as NSG mice, allows researchers to reconstitute the immune system of these mice with human immune cells, providing an experimental platform to investigate atherosclerotic mechanisms. Observing how the immune cells drive disease in these animals may better recapitulate what occurs in human atherosclerosis. Furthermore, employing multi-omics approaches to study HIV-associated atherosclerosis in humanized mouse models provides a means to gather a comprehensive understanding of the similarities and differences in molecular mechanisms between the two species, which enhances the value of mouse models in cardiovascular research. Specifically, humanized mouse models provide a means to significantly enhance our capacity to address critical gaps in understanding the immune mechanisms driving HIV-associated atherosclerosis.

## Figures and Tables

**Table 1:** Selecting the ideal mouse model for atherosclerosis research.

Mouse Model	Research Focus
*Apoe*^−/−^ mice	Spontaneous atherosclerosis development Role of ApoE in atherosclerosis Inflammation and atherosclerosis
CETP.E3L mice	Role of age-related changes in cholesterol transport and lipid profiles in atherogenic processes
*Ldlr*^−/−^ mice	Diet-induced atherosclerosis Role of LDL receptor in cholesterol handling and atherosclerosis Interactions between diet and genetic differences in human LDL receptor Human-like lipid profile and atherosclerosis (for example to study the role of statins) Interaction between diet and inflammation
*Apoe*^−/−^ *Fbn1*^C1039G+/−^ mice	Arterial stiffening and plaque development
Tg26^+/−^/*Apoe*^−/−^ mice	Role of HIV transcripts and proteins in atherosclerosis development and progression

**Table 2:** Limitations of mouse models: summary of differences in atherosclerosis between mice and humans.

Aspect	Mice	Humans
Cholesterol Transport	Transport most cholesterol in HDL particles (atheroprotective).	Transport most cholesterol in LDL particles (atherogenic).
Bile Acid Metabolism	Produce more hydrophilic bile acids, leading to reduced intestinal cholesterol uptake and increased fecal cholesterol excretion.	Produce less hydrophilic bile acids, leading to higher intestinal cholesterol absorption.
Atherosclerotic Plaque Location	Plaques develop in the brachiocephalic trunk, aortic arch, aortic sinus, and proximal aorta.	Plaques typically develop in the carotid and coronary arteries.
Plaque Development	Plaques develop but do not progress to advanced fibrous atheroma.	Plaques progress to advanced-stage fibrous atheroma.
Genetic Models	Common models include *Apoe^−^*^/^*^−^* and *Ldlr^−^*^/^*^−^* mice, manipulated for hypercholesterolemia.	Genetic mutations in *Ldlr* and *Apoe* are associated with familial hypercholesterolemia and ASCVD.
Plaque Rupture	Plaque rupture is rare except in specific models (e.g., *Apoe*^−/−^ *Fbn1*^C1039G+/−^ mice, angiotensin II-infused *Apoe^−^*^/^*^−^* mice).	Plaque rupture is common, leading to thrombosis and cardiovascular events.
Inflammatory Response	Th1-skewed immune response; IFN-γ plays a key role in lesion development.	Mixed Th1/Th2 immune response; inflammation plays a significant role in plaque rupture.

## Abbreviations

The following abbreviations are used in this manuscript:

APOE	apolipoprotein E
ART	antiretroviral therapies
ASCVD	atherosclerotic cardiovascular disease
CAD	coronary artery disease
CAMs	cell adhesion molecules
cART	combination antiretroviral therapy
CETP	cholesteryl ester transfer protein
CMV	cytomegalovirus
CRISPR/Cas9	clustered regularly interspaced short palindromic repeats/CRISPR-associated 9
CVD	cardiovascular disease
DCs	dendritic cells
DKO	double knockout
DOI	DNA of interest
dsRNA	double-stranded RNA
ECM	extracellular matrix
ECs	endothelial cells
eNOS	endothelial nitric oxide synthase
eQTLs	expression quantitative trait loci
ER	endoplasmic reticulum
Fbn1	fibrillin 1
gp	glycoprotein
GWAS	genome-wide association studies
HDL	high-density lipoprotein
HEs	homing endonucleases
HIV	human immunodeficiency virus
HSC	hematopoietic stem cell
IFN	interferon
INSTI	integrase strand transfer inhibitors
KO	knockout
KTR	kynurenine to tryptophan ratio
LDL	low-density lipoproteins
LDLR	low-density lipoproteins receptor
MDM	monocyte-derived macrophage
MHC	major histocompatibility
M-tropic	macrophage-tropic
mRNA	messenger RNA
NADPH	nicotinamide adenine dinucleotide phosphate
NF-kB	nuclear factor kappa B
NHEJ	non-homologous end joining
NK	natural killer
NO	nitric oxide
NOX	nicotinamide adenine dinucleotide phosphate oxidase
NNRTI	non-nucleoside reverse transcriptase inhibitors
NRTI	nucleoside reverse transcriptase inhibitors
NSG	NOD scid gamma
ox-LDL	oxidized-LDL
PBMC	peripheral blood mononuclear cells
PI	protease inhibitors
PITT	pronuclear injection-based targeted transgenesis
PI3K	phosphoinositide 3-kinase
PLWH	people living with HIV
PWoH	people without HIV
RISC	RNA-induced silencing complex
RMCE	recombination-mediated cassette exchange
RNA	ribonucleic acid
RNAi	RNA interference
ROS	reactive oxygen species
siRNA	small interfering RNAs
TALENs	transcription activator-like effector nucleases
Tat	trans-activator of transcription
TREM	triggering receptor expressed on myeloid cells
UPR	unfolded protein response
VSMC	vascular smooth muscle cell
ZFNs	zinc-finger nucleases
